# Spontaneous Pneumothorax in a Healthy Young Woman: Discussion About Treatment Options

**DOI:** 10.7759/cureus.55633

**Published:** 2024-03-06

**Authors:** Leah Brill, Nina Li, Gerardo Carino

**Affiliations:** 1 Biology, College of Arts and Sciences, University of Vermont, Burlington, USA; 2 Pulmonary and Critical Care, Warren Alpert Medical School at Brown University, Providence, USA

**Keywords:** secondary pneumothorax, primary spontaneous pneumothorax, video-assisted thoracic surgery (vats), pleural blebs, chest tube

## Abstract

A spontaneous pneumothorax, a potentially life-threatening condition, is a disease process in which air enters the space between the visceral and parietal pleural of the lung, thus increasing the pressures in that space. It can be diagnosed by both physical exam and radiographic testing. In this case, we present a 21-year-old, otherwise healthy woman who presented with sudden, sharp shoulder pain and chest tightness and was diagnosed with her first, spontaneous pneumothorax. We further discuss the diagnosis and treatment options for a first-time spontaneous pneumothorax.

## Introduction

A pneumothorax is a collection of air outside the lung, which accumulates between the visceral and parietal pleura, a space defined as the pleural space. The pleural space usually maintains a negative intrapleural pressure, relative to pressure of 0 cm of water in both the lungs and thoracic cavity [1]. However, air from a pneumothorax in this space increases the pressure which may lead to lung collapse and severe hemodynamic compromise, which can be life-threatening. A case in which there is no clear inciting event or cause is known as spontaneous pneumothorax.

The degree of collapse of the lung determines the presentation of a pneumothorax. There are multiple types of pneumothoraces: spontaneous, traumatic, and tension. Spontaneous pneumothorax is when there is no clear inciting event or cause, such as in the case being presented. Spontaneous pneumothorax is further divided into primary spontaneous pneumothorax (PSP) and secondary spontaneous pneumothorax (SSP). Primary spontaneous pneumothorax occurs without an apparent cause or underlying lung disease. Risk factors include tall thin adolescent males, smoking, especially marijuana smoking, pregnancy, and Marfan Syndrome. Primary spontaneous pneumothoraces are more common in men than women, and their prevalence is 77 cases per 100,000 hospital visits in the United States [2]. 

Secondary spontaneous pneumothorax (SSP) occurs in patients with an underlying lung disorder. Diseases associated with secondary spontaneous pneumothorax include asthma, cystic fibrosis, chronic obstructive pulmonary disease (COPD), inhalation drug use, lung cancer, and interstitial lung disease (ILD) [3]. Any bullae that form in the lung, either acutely or chronically, may increase the risk of pneumothorax. The rupture of these bullae can lead to a leak in the visceral pleura, allowing air to accumulate from the airway into the pleural space. Secondary spontaneous pneumothoraces generally occur in older patients compared to primary spontaneous pneumothoraces, and are more likely to develop persistent air leaks, due to the underlying lung disease; they also have a higher recurrence risk when compared to secondary spontaneous pneumothoraces [4]. 

## Case presentation

The patient is a 21-year-old college student who presented to the hospital after experiencing an acute onset of sharp shoulder and scapular pain and a feeling of "something squeezing her heart" that awoke her from sleep. The chest pain was not associated with shortness of breath, fever, chills, or cough. She denied any prior history of lung disease, smoking, and marijuana use but did have some secondhand smoke exposure. She had traveled on a six-hour flight three weeks earlier and did not participate in any scuba diving, climbing, or Valsalva maneuvers as described to her. She also denied nausea, vomiting, abdominal pain, urinary symptoms, and headache and her last period had been normal four days prior. She did report similar symptoms a year prior which resolved on their own without a workup or intervention.

**Figure 1:**
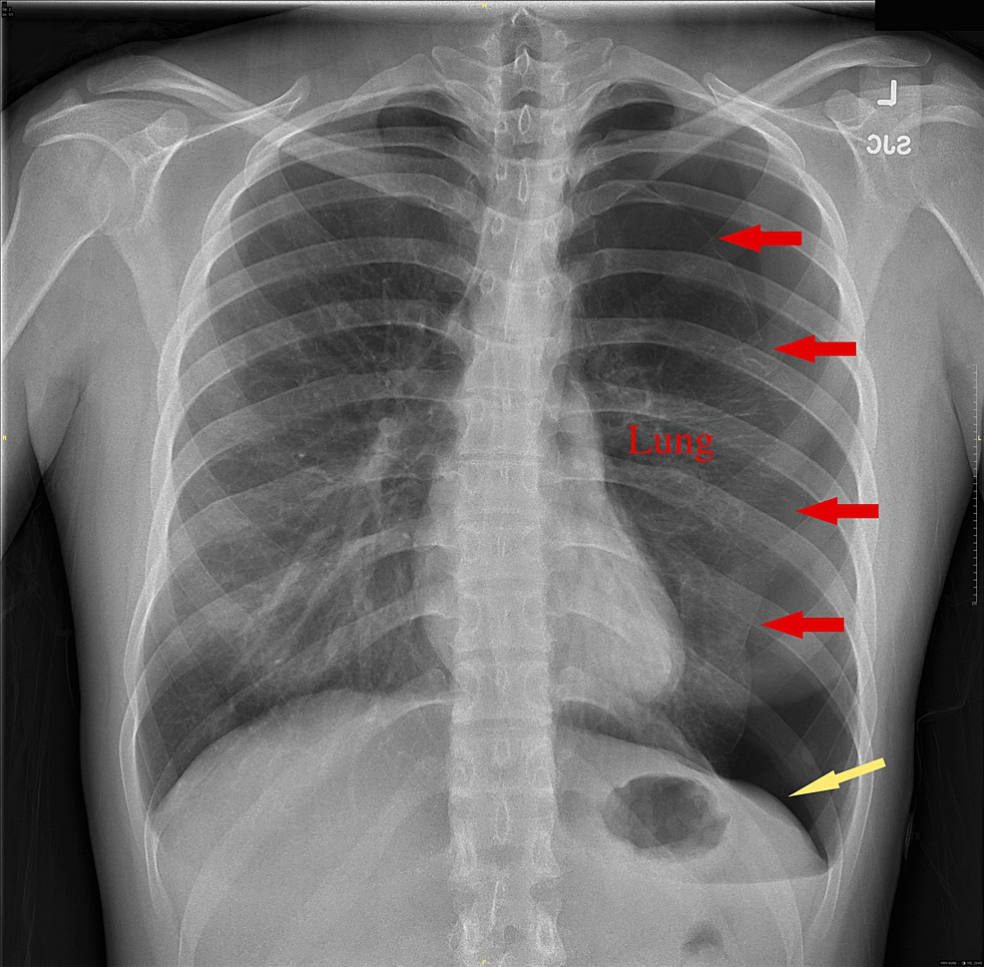
Chest x-Ray on presentation showing a left-sided pneumothorax, marked by red arrows. There is also a mild lowering of the left diaphragm (a deep sulcus sign, yellow arrow).

On physical exam, she was well nourished and well developed in no apparent distress. Diminished breath sounds without wheezing or rhonchi were noted on the left. Laboratory analysis was unremarkable. A chest radiograph showed a large left-sided pneumothorax (Figure 1), approximately 50% of her thoracic volume with an associated mild deep sulcus sign. The patient’s vital signs were 98% on room air, a blood pressure of 102/65 mmHg, a heart rate of 65, a respiratory rate of 16, and a temperature of 36.1 Celsius.

**Figure 2:**
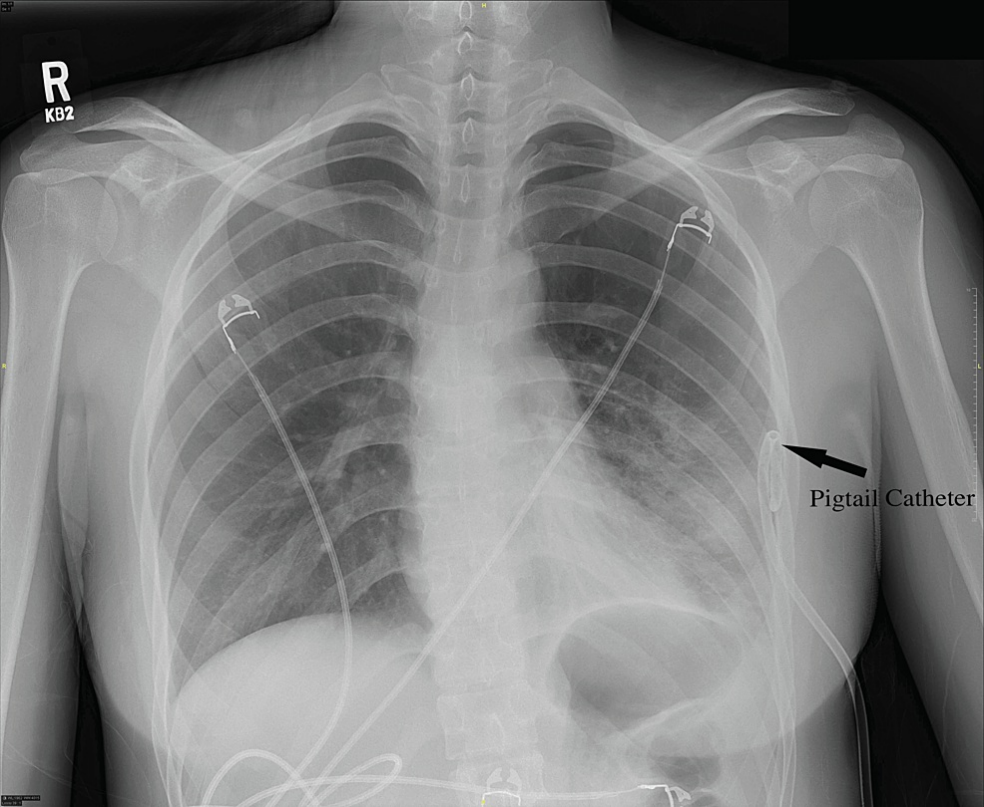
Re-expansion of the pneumothorax after placement of a small-bore pigtail catheter.

A small-bore, percutaneous pigtail catheter was placed in the emergency department and the patient’s chest tightness improved. A follow-up chest x-ray showed a well-positioned chest tube with a resolution of the pneumothorax (Figure 2). She was admitted to the hospital and maintained on 20 cm of suction overnight. The next day, the chest tube was placed on a water seal but a chest x-ray then showed a recurrence of the pneumothorax, so suction was re-initiated. The following day, again, the pneumothorax recurred on the water seal, so a chest CT was obtained. The CT demonstrated a small remaining apical pneumothorax and the chest tube in position. A small bleb was also noted in the left apex. 

Thoracic surgery was consulted as the pneumothorax failed to resolve despite multiple conservative attempts. Due to the presence of the bleb, the decision was made to proceed to surgery and the patient underwent left-sided, video-assisted thoracoscopy (VAT) with blebectomy and mechanical pleurodesis. She did well post-procedure; the chest tube was able to be removed post-op day three and she was discharged home. She is currently doing well months after her procedure.

## Discussion

A spontaneous pneumothorax should be considered in any patient who presents with the acute onset of shortness of breath and pleuritic chest pain, especially in patients with the risk factors of young age (often 20-40 old) and tall, thin stature [5]. Sharp pain radiating to the ipsilateral shoulder is also common. Spontaneous pneumothorax is more common in men and smokers, including marijuana, and individuals with underlying lung disease. Any recent activities that may result in large shifts in intrathoracic pressure, like flying at altitude, mountain climbing, or scuba diving can also increase the risk of pneumothorax. Genetics may also play a role. 

Menstruation-related spontaneous or catamenial pneumothorax (CP), while rare, has been demonstrated to be a significant cause of spontaneous pneumothorax in women of reproductive age. Of 114 women who underwent VATS for treatment of spontaneous pneumothorax at a medical center over six years, 24.6% had catamenial pneumothorax [6]. CP is defined as a spontaneous pneumothorax occurring within 72 hours before or after the onset of menstruation with histologic examination of pleural tissue demonstrating endometriosis [6]. While there is no clear explanation for the migration of endometrial cells to the thoracic cavity and the resulting pneumothorax, management requires collaboration between thoracic surgeons and gynecologists due to the high recurrence rate despite surgical intervention [7]. The patient had reported the ending of menses four days before her admittance and subsequent histologic examination did not find endometriosis, suggesting this was not a case of CP and instead a PSP. 

Often diagnosis can be made very simply with a chest radiograph; however, the findings of a small pneumothorax can be subtle and missed [8]. Point-of-care ultrasound can also diagnose pneumothorax but is dependent on the skill set of the sonographer. In skilled hands, ultrasonography has been shown to have an accuracy of 85% in detecting pneumothorax, while chest radiography has an accuracy of 80% with a sensitivity of only 60% [9]. Chest CT is more commonly used as the gold standard to diagnose pneumothorax in inpatient settings and has the advantage of providing information about the underlying lung parenchyma. 

Treatment of primary spontaneous pneumothorax is mainly dependent on the size of the pneumothorax and the stability of the patient, with the main treatment goal being the evacuation of air from the pleural space to allow the healing of any injury. In a stable patient with a small pneumothorax, usually defined as less than 2-3 cm in distance from the pleural space, the pneumothorax may heal on its own without intervention. These patients can generally be observed with supplemental oxygen by nasal cannula. The supplemental oxygen helps with the resorption of any nitrogen in the pleural space. If observed for four to six hours (often, in practice, overnight), and there is no evidence of worsening of the pneumothorax on imaging, the patient can generally be discharged with recommendations to return with any worsening symptoms [10]. This approach may also be considered with very small ( > 1cm) secondary spontaneous pneumothorax with limited symptoms; however, there is likely a higher need for intervention in these cases [11]. 

Aspiration can be used to reduce the size of the primary spontaneous pneumothorax and according to some studies, may be as effective as a chest tube as a first-line intervention. In a recent meta-analysis of 4,262 patients with a first-time primary spontaneous pneumothorax, aspiration was found to be as effective as chest tube drainage [12]. Needle aspiration is done by inserting a needle into the intrapleural space, and air is aspirated to achieve re-expansion of the lung. The British Thoracic Society (BTS) guidelines suggest that a maximum of 2.5 liters can be removed [13]. 

In practice, a chest tube is the most commonly used tool that removes air from the pleural space and reestablishes the negative pressure in the chest cavity. Chest tubes can be placed as the first line, if needle aspiration fails or in any case with possible tension physiology. Chest tubes are placed between the fourth and fifth intercostal space at the mid-axillary line [14]. While 28-32 French sizes are widely used, the size of the chest tube has been shown not to affect the drainage, rate of complications, or need for additional tube drainage [15]. Ultrasound may be used to guide the placement of the tube. An x-ray can be used to confirm re-expansion of the lung and the chest tube can be removed once air leaking stops. 

Recognition of a tension pneumothorax, where the pneumothorax results in displacement and compression of mediastinal structures, is crucial and should be followed by immediate intervention. Delayed recognition increases the mortality rate, reaching up to 91% when compared to 3%-7% if recognized early [16]. The patient should be given high-concentration oxygen, aspiration, or cannula for decompression, and a chest tube should be inserted into the pleural space as soon as possible [10,17]. 

The use of a small-bore pigtail catheter (PC) versus a large-bore, surgically-placed, chest tube (LBCT) as the initial treatment for pneumothorax is still subject to debate, resulting in variations in clinical practice. A meta-analysis demonstrated a similar success rate in PC (79.8%) and LBCT (82.9%) for all pneumothorax types, and no significant difference was observed in intervention success rate when analyzed according to pneumothorax types. However, the PC group did have significantly lower complication rates and hospital stays than the LBCT group [12]. Many centers have moved to PC as the primary intervention over larger chest tubes.

The possibility of recurrence is at its highest within the first six months to two years after the initial pneumothorax. The recurrence rate of primary spontaneous pneumothorax has been found to be around 54% within the first four years, with an increased risk in individuals who smoke and who are tall and thin. Risk factors for greater recurrence in secondary spontaneous pneumothorax include age and any underlying lung disease [17]. Another study demonstrated a 26.7% recurrence within the first seven months after primary spontaneous pneumothorax treated with a chest tube [18]. The risk of recurrence increases with each recurrent pneumothorax. For 25-50% of patients with secondary spontaneous pneumothorax, surgical intervention is needed due to persistent air leakage, discovery of underlying parenchymal disease, or recurrence [19]. Recurrence rates after VATS treatment dramatically decrease, ranging from 0 to 11% with different pleurodesis techniques, and risk factors for postoperative recurrence include older age (<40 years), smoking, underlying lung condition, and prolonged postoperative air leakage [20].

## Conclusions

Pneumothorax is a common problem physicians face, so knowing the proper diagnosis and treatments is necessary. The chest x-ray is the most commonly used tool to diagnose pneumothorax in hospital settings, although the use of ultrasonography is on the rise due to its high accuracy level. Needle aspiration and placement of a chest tube are two techniques used to re-expand the lung and have shown similar results. Recurrence is a common side effect, especially among secondary spontaneous pneumothorax and in individuals who smoke. Persistent air leakage, recurrence, or other underlying causes should be considered when deciding if surgical consultation and VATS will be pursued. It is also important to follow up with the healthcare provider post-pneumothorax to aid in rehabilitation and safe return to activities.
